# An exploratory study assessing psychological distress of indigents in Burkina Faso: a step forward in understanding mental health needs in West Africa

**DOI:** 10.1186/s12939-017-0633-8

**Published:** 2017-08-14

**Authors:** Émilie Pigeon-Gagné, Ghayga Hassan, Maurice Yaogo, Valéry Ridde

**Affiliations:** 1 Department of Psychology, Université du Québec à Montréal, Montreal, Canada; 2 Département Lettres et Sciences Humaines (LSH), Université Catholique de l’Afrique de l’Ouest, Unité Universitaire à Bobo-Dioulasso (UCAO-UUB), Bobo-Dioulasso, Burkina Faso; 3 Department of Social and Preventive Medicine, University of Montreal School of Public Health (ESPUM), Montreal, Canada; 4 University of Montreal Public Health Research Institute (IRSPUM), Montreal, Canada

**Keywords:** Global mental health, Transcultural psychiatry, Poverty, Psychological distress, Psychotic symptoms, Indigence, West Africa, Mental health needs

## Abstract

**Background:**

Poverty is known as an important determinant of health, but empirical data are still missing on the relationships between poverty, other adverse living conditions, and psychological distress, particularly in low-income countries.

This study aimed to assess mental health needs and psychological distress among the poorest in rural settings in Burkina Faso where food security and access to water, electricity, schooling, and healthcare are limited.

**Methods:**

We randomly selected 2000 individuals previously identified as indigents by a community-targeting process. Interviewers visited participants (*n* = 1652) in their homes and completed a questionnaire on mental health variables that included presence and intensity of anxious, depressive, psychotic, and aggressive symptoms, as well as level of psychological distress. Descriptive statistics, Spearman correlations, and logistic regressions were performed.

**Results:**

In all, 40.2% of the sample reported 10 or more anxious/depressive symptoms in the past 30 days, and 25.5% reported having experienced at least one psychotic symptom over their lifetime, 65.6% of whom had had those symptoms for many years. The number of anxious and depressive symptoms was significantly associated with the level of psychological distress (*r* = 0.423, *p* < .001). Predictors of distress level included: poor health condition (F(1) = 23.743, *p* <. 001), being a woman (F(1) = 43.926, *p* < .001), not having any income (F(1) = 16.185, *p* < .001), having begged for food in the past 30 days (F(1) = 12.387, *p* < .001), being illiterate, and being older (F(1) = 21.487, *p* < .001). Approximately one third of respondents reporting anxious/depressive or psychotic symptoms (28.2 and 30.0%, respectively) had not talked about their symptoms to anyone in their social network.

**Conclusions:**

These results suggest alarmingly high levels of psychological distress and reported symptoms among the poorest in rural settings in Burkina Faso, which can be explained by their difficult living conditions. However, these results must be interpreted from a transcultural perspective to avoid decontextualized misinterpretations. Ethnographic works are needed to document the larger context within which these distress results can be analyzed.

## Background

The World Health Organization defines mental health as “a state of well-being in which every individual realizes his or her own potential, can cope with the normal stresses of life, can work productively and fruitfully, and is able to make a contribution to her or his community” [1]. Promoting mental health is increasingly becoming a global public health issue, partly because a large number of people worldwide are unable to live in a healthy mental state. Indeed, it is estimated that mental and substance disorders represent the fifth leading cause of global disability-adjusted life years (7.4%), and the leading cause of years lost due to disability (22.9%) [2]. Furthermore, in some low and middle-income countries, between 70% and 90% of mentally ill individuals have no access to care [3]. Addressing this gap in access to services and providing adequate treatment to individuals suffering from mental health disorders in resource-limited settings have recently been recognized as global health priorities [4].

The literature on social determinants of health indicates that living in chronic poverty is associated with a higher risk of developing various psychopathologies [5–7]. Evidence suggests that some indicators of poverty (i.e., poor or inexistent access to education, food insecurity, and living in an unstable environment) are linked to the risk of developing common mental disorders across settings, while other indicators show inconsistent results, partly because they are highly dependent on social, political, and cultural contexts [8, 9, 10]. The lack of empirical studies on mental health in low-income countries limits the current understanding of the mechanisms underlying the relationships between poverty, poor living conditions, and mental health outcomes in specific contexts.

For the current study we focused on Burkina Faso, one of the poorest countries in the world, with 73% of the population living in rural settings where food security and access to water, electricity, schooling, and healthcare - including mental health care - are reduced [11]. Rural communities depend largely on agriculture and farming to meet their needs [12]. In these areas, traditional gender roles prevail, with women constituting a socio-economically vulnerable group, particularly the elderly or widowed [13].

It is estimated that 40.1% of Burkina Faso’s population lives below the national poverty line [14] and that around 9% of the population lives below the extreme poverty line (annual income of less than 100 US$) and are identified as indigents [15, 16]. In the past decade, indigents have been identified in rural Burkina Faso through a community-based approach [17–19] in which various health professionals and community authorities were interviewed to choose criteria for an indigent selection process. Poor mental health and the presence of psychopathologies were unanimously recognized as being important indicators of vulnerability for indigence [20]. Despite this community insight, no previous empirical studies in West Africa have investigated mental health indicators among indigents [21], even though they are a group with specific health needs and less access to health care than the general population [18, 22, 23].

Besides the absence of evidence on indigents’ mental health, little is known about psychological distress in Burkina Faso. Only a few studies have been conducted in the capital city among clinical populations affected by various conditions such as HIV [24], dementia [25], depressive disorders [26–29], and transitory psychotic disorders [30]. These studies inform on the prevalence of specific conditions but provide little information on the development of pathologies and the influence of poverty and living conditions on psychological distress.

As Ouédraogo and colleagues [31] have pointed out, people seeking help from psychiatric facilities are those for whom traditional medicine has not worked and who have developed a chronic or recurrent condition (transitory psychotic disorder, schizophrenia, or major depressive disorder). They are thus not representative of the overall mental health needs of the general population, who are more likely to consult traditional healers due to greater compatibility with their beliefs [32]. Not only does the modern medical system not correspond to local conceptions of mental illness, but services also remain poorly accessible outside of urban settings [33].

We found only two studies conducted in Burkina Faso with a focus on a non-clinical sub-population. Both highlight that poverty and poor living conditions are linked to the risk of experiencing psychological distress. The qualitative study by Nanama and Frongillo [34] conducted in a rural area in the north of the country showed how food insecurity could be related to a high level of psychological distress and to certain anxious, depressive, and somatic symptoms. The study by Duthé and colleagues [35], conducted in the peripheral neighbourhoods of Ouagadougou, found that chronic illnesses or handicaps, extreme poverty, and food insecurity predicted the risk of developing a depressive disorder among their participants.

### Aim of the study

Considering the absence of empirical studies on the mental health needs of indigents in Burkina Faso, our study aimed to document the ways in which psychiatric symptoms are expressed, as well as the predictors associated with psychological distress among this vulnerable sub-population. More specifically, our study aimed to gather data on: 1) the prevalence of anxious, depressive, and psychotic symptoms, 2) the help-seeking behaviours associated with these symptoms, and 3) the factors leading individuals to experience a high level of psychological distress.

## Methods

### Sample

In 2014, a performance-based financing intervention was implemented in 10 health districts in Burkina Faso, where an indigent selection process was conducted whose effectiveness had previously been demonstrated in one district [17]. This process started at the village level where, in each village, a committee composed of local authorities identified indigent individuals on the basis of the following definition: “a person who is extremely socially and economically disadvantaged, unable to take care of himself (herself), and without any internal or external resources” [36]. Each village’s list of indigents was then transmitted to the closest health centre, where a committee was formed to ensure follow-up and verify that the selection had been done appropriately. The lists provided by the health centre level committees were then validated at the health district level. Of the 1,032,541 inhabitants of the four health districts of interest (Diébougou, Gourcy, Kaya and Ouargaye) 51,267 were identified as indigents [36].

For the current study, a stratified sampling procedure was employed. At the district level, four districts were randomly selected from the 10 districts where the targeting process had been implemented. In Burkina Faso, every health district contains multiple communes, themselves containing multiple villages. We randomly selected communes in each district, and from those we randomly selected villages. We only included in the sampling procedure villages where at least 10 individuals had been identified as indigents. From the selected villages, we interviewed every individual over 18 years old whose name was on the original list. This stratified procedure produced a sample of 2000 indigents from the 51,567 in the original lists across the four districts.

### Data collection

The data were collected between February 24, 2015, and April 30, 2015. Trained interviewers visited indigents in their homes. After obtaining verbal consent, the interviewer questioned the individuals and their families in local languages. The results presented in this article focus on the data obtained from the individual questionnaires regarding socio-demographic information, health services use, health condition, mental health needs, and cognitive functioning. The questionnaire was administrated with ODK software on smartphones from which data was transferred daily to the main database for regular check-ups.

### Outcomes and variables

To our knowledge, there is no valid tool for assessing mental health in West Africa. To create the questionnaire for the current study, we referred to the following instruments: the Refugee Health Screener (RHS-15), the Kessler Psychological Distress Scale (K-10) and the Composite International Diagnostic Interview (CIDI). The RHS-15 is a screening tool used for refugees to assess, in a culturally sensitive manner, their level of psychological distress and the presence of mental disorders. The K-10 is a 10-item questionnaire that assesses anxiety and depression. The CIDI is a comprehensive questionnaire with 42 sections, each measuring the presence and intensity of a psychopathology. All three instruments were originally designed and used for clinical and research purposes. The latter two instruments have been widely used in previous epidemiologic studies. It should be noted that these standardized tools were originally built from Western scientific literature and diagnosis criteria (DSM-IV-TR), which limits their validity in transcultural contexts. Because none of these tools was totally appropriate in the current study context, we constructed one by selecting relevant questions, which we then reformulated and adapted to fit the socio-cultural context, the local language, and the documented idioms of distress [37]. The questionnaire was reviewed by an anthropologist (MY) living in Burkina Faso who worked with the cultural groups targeted by this study and had been involved in former studies about the worst-off with VR [17, 38]. Following that review, the questionnaire was pre-tested and interviewers trained.

The final instrument contained 28 questions: 14 on anxious and depressive symptoms (within the past month) (α = .915), five on psychotic symptoms (over the lifetime) (α = .865), three on aggressive behaviours (over the lifetime) (α = .520), two on help-seeking behaviours directed towards formal and informal resources (“Have you talked about this (or these) symptom(s)?”, “To whom have you talked about this (or these) symptom(s)?”), one on general psychological distress (“In general, do you feel you are able to cope with difficulties that comes in your way?”), one on functional impairment due to the presence of anxious or depressive symptoms (“To what point do these symptoms limit your ability to perform your daily tasks?”), one on the duration of psychotic symptomatology, and one on the intensity of psychotic symptomatology. Given the absence of clinical norms and because this tool was used for an exploratory purpose, no cut-offs were determined; instead we used continuous scores in a correlational design.

The following variables were also assessed: age, sex, marital status, schooling level, literacy, perceived health status, presence of handicaps, cognitive functioning (measured by the Leganés cognitive test), presence of income, ability to fulfill nutritional needs, and presence of begging behaviours to obtain food from other community members.

### Statistical analysis

SPSS21 software was used to perform descriptive and Spearman correlations, as well as binary and multinomial logistic regressions. In these analyses, the 14 anxious or depressive symptoms, the five psychotic symptoms, and the three aggressive manifestations were used as binary variables (presence or absence). For each regression the following were used as independent variables: age (continuous variable), sex (male, female), marital status (single, monogamous, polygamous, widowed, separated/divorced), perceived health status (poor, average, good), altered cognitive functioning (yes, no), literacy (yes, no), begging behaviours (yes, no), fulfilled nutritional needs (yes, no), income (yes, no), anxious or depressive symptoms (yes, no), psychotic symptoms (yes, no), and aggressive behaviours (yes, no). For the binary regression, the dependant variable used was the presence/absence of help-seeking behaviours among individuals who reported any symptom. For the multinomial regression, the dependant variable used was the level of psychological distress. For this analysis, the five-level psychological distress variable was changed to a three-level variable (low, medium, high level of distress) to simplify the analysis and to have a minimal number of respondents per group. This change had no significant impact on the results.

## Results

From the original sample of 2000 selected indigents living in the four health districts of interest, 348 could not participate because of physical absence (6.9%), mental or physical handicap (3.9%), illness (1.3%), advanced age (1.9%), or death (1.1%). Of the 1652 (83%) who participated, 1117 were women (67%) and 535 men (33%). Individuals were aged between 18 and 98 years, with a mean age of 55 years. Other socio-demographic characteristics of the sample are presented in Table 1.

**Table 1:** Socio-demographic and descriptive characteristics of indigents in the sample

Variables		*n*	%
Sex	Female	1117	67.6
	Male	535	32.4
Age	18–24 years	79	4.8
	25–49 years	485	29.4
	50–64 years	509	30.8
	65 and older	579	35.0
Marital status	Married, monogamous	564	34.1
	Married, polygamous	410	24.8
	Widowed	461	34.0
	Single	78	4.7
	Separated/divorced	39	2.4
Schooling	No	1561	94.5
	Yes	91	5.5
Literacy	No	1548	93.7
	Yes	104	6.3
Income	No	1435	86.9
	Yes	217	13.1
Perceived health status	Good	322	19.5
	Average	891	53.9
	Poor	439	26.6
Handicap	No	1263	76.5
	Yes	389	23.5
Cognitive functioning	Altered	1095	66.3
	Non-altered	557	33.7
Ability to fulfill nutritional needs	No	885	53.6
	Yes	767	46.4
Begging behaviours	No	1364	82.6
	Yes	288	17.4

### Anxious and depressive symptomatology

With regard to the 14 anxious or depressive symptoms covered by the questionnaire, 11.6% of respondents reported not having experienced any in the past month, 17.9% reported one to four symptoms, 30.3% reported five to 10 symptoms, and 40.2% reported more than 10. Table 2 shows the proportions of self-reported anxious and depressive symptoms.

**Table 2:** Proportions of self-reported anxious and depressive symptoms^a^

Symptoms	*n*	%
Feeling a profound sadness	1237	74.9
Thinking too much or having too many thoughts	1204	72.9
Being exhausted or lacking energy without any reason	1162	70.3
Being constantly worried	1109	67.1
Not being able to sleep at night (insomnia)	1076	65.1
Feeling hopeless or helpless	1003	60.7
Experiencing very fast or very strong heartbeats	900	54.5
Feeling stuck with an unsolvable problem	840	50.8
Experiencing dizziness or weakness	804	48.7
Having burning sensations in your body	763	46.2
Experiencing heart pain or a pressing sensation on your heart	721	43.6
Feeling you have worms crawling in your head	515	31.2
Feeling so nervous than nothing could calm you down	460	27.8
Lacking motivation to visit your relatives or be with them	399	24.2

^a^In the past month

Of the individuals who reported experiencing at least one of the 14 anxious or depressive symptoms, 32.9% reported that this did not affect their capacity to carry on their usual daily activities, while 25.6% reported that their symptoms slightly affected their regular functioning and 41.5% said their symptoms greatly affected their functioning.

The total number of anxious and depressive symptoms reported by participants was significantly correlated to their level of functioning (*r* = .423, *p* < .001, *n* = 1460). Each anxious and depressive symptom was individually correlated to the level of functioning, except for the *lacking motivation to visit your relatives or be with them* item. However, none of the symptoms seemed to be, in and of itself, a significant predictor of the level of functioning reported by the respondents.

### Psychotic symptomatology

Our results revealed that 25.5% of the sample reported having experienced at least one psychotic symptom, and 13.1% reported aggressive behaviours over their lifetime. Of the 421 individuals who had experienced at least one psychotic symptom, 65.5% reported having had this (or these) symptom(s) for many years, which may reflect either a high prevalence of psychopathology or a culturally relevant way of expressing distress without the existence of a psychotic diagnosis. Table 3 shows the proportions of self-reported psychotic symptoms and agressive manifestations.

**Table 3:** Proportions of self-reported psychotic symptoms^a^ and aggressive behaviours^b^

Symptoms	*n*	%
Having the sensation of being possessed by some mysterious force or by a spirit	284	17.2
Having the sensation of being outside your body	265	16.0
Hearing things that other people cannot hear	241	14.6
Seeing things that other people cannot see	194	11.7
Being so mad you feel out of control	188	11.4
Feeling that people, objects, or places around you are not real	180	10.9
Feeling the urge to hurt yourself	47	2.8
Feeling the urge to hit, push, or hurt someone around you	46	2.8

^a^In the past month

^b^Over the lifetime

### Help-seeking behaviours

There were no significant differences in terms of help-seeking behaviours for psychotic and depressive symptoms. In both cases, the majority of respondents had talked about their symptoms with a family member, and less than 5% had sought help from a health-care professional or a traditional healer (Table 4).

**Table 4:** Help-seeking behaviours for anxious/depressive and psychotic symptoms

	Anxious or depressive symptoms		Psychotic symptoms	
	*n*	%	*n*	%
Family member	981	67.2	270	64.3
Health-care professional	25	1.7	3	0.7
Traditional healer	22	1.5	12	2.9
Friend	20	1.4	7	1.7
No one	412	28.2	128	30.5
Total	1460	100.0	420	100.1

A binary logistic regression (Table 5) revealed that people reporting the following characteristics more significantly more at risk of not seeking having sought help: having a poor (F(1) = 25.991, *p* < .001) or average (F(1) = 15.166, *p* < .001) perceived health status, begging for food (F(1) = 17.412, *p* < .001), and not having any income (F(1) = 4.059, *p* = .044).

**Table 5:** Predictors of the absence of help-seeking behaviours

Parameters		*n*	B	Std. Error	Hypothesis Test		Exp(B)	95% Wald Confidence	
					Wald Chi-Squared	Sig.		Lower	Upper
Threshold	Absence vs. presence of help-seeking behaviours		−1.149	.3280	12.277	.000	.317	.167	0.603
Sex	Male	535	−.223	.1491	2.229	.135	.800	.598	1.072
	Female	1117	0^a^				1		
Literacy	Yes	104	.175	.2737	.407	.523	1.191	.696	2.036
	No	1548	0^a^				1		
Marital status	Separated/divorced	39	.967	.5007	3.733	.053	2.631	.986	7.021
	Single	78	.057	.3110	.034	.853	1.059	.576	1.948
	Married, polygamous	410	.035	.1616	0.47	.828	1.036	.755	1.422
	Widowed	561	−.199	.1724	1.326	.249	.820	.585	1.150
	Married, monogamous	564	0^a^				1		
Income	Yes	217	−.344	.1710	4.059	.044	.709	.507	.991
	No	1435	0^a^				1		
Handicap	Yes	389	.152	.1551	.959	.327	1.164	.859	1.578
	No	1263	0^a^				1		
Nutritional needs fulfilled	Yes	767	.084	.1237	.461	.497	1.088	.853	1.386
	No	885	0^a^				1		
Begging behaviours	Yes	288	.761	.1823	17.412	.000	2.140	1.497	3.059
	No	1364	0^a^				1		
Perceived health status	Good	322	−.854	.2194	15.166	.000	.426	.277	.654
	Average	891	−.809	.1587	25.991	.000	.445	.326	.608
	Poor	439	0^a^				1		
Altered cognitive functioning	No	1095	.118	.1401	.714	.398	1.126	.885	1.481
	Yes	557	0^a^				1		
Age		1652	.005	.0045	1.351	.245	1.005	.996	1.014

^a^Set to 0 because this parameter is redundant

### Psychological distress

**Fig. 1:**
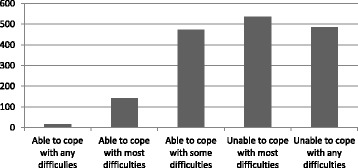
Distribution of the number of participants (*n* = 1652) reporting psychological distress as measured by the perceived ability to cope with life difficulties

Figure 1 presents the sample distribution with respect to their perceptions of being able (or not) to handle difficulties encountered in their life. This five-level variable was used as an indicator of psychological distress and showed a significant correlation with the total number of anxious and depressive symptoms (*r* = .243, *p* < .001, *n* = 1652), as well as with the total number of psychotic symptoms reported by the respondents (*r* = .113, *p* < .001, *n* = 1652).

As shown in Table 6, the following variables were significant predictors of respondents’ level of distress (three-level variable: low, medium, and high level of distress): being a woman (F(1) = 44.247, *p* < .001), reporting at least one anxious or depressive symptom (F(1) = 41.557, *p* < .001), having a poor (F(1) = 26.614, *p* < .001) or average (F(1) = 23.233, *p* < .001) perceived health status, being single (F(1) = 21.244, *p* < .001), being elderly (F(1) = 19,045, *p* < .001), having no income (F(1) = 15,578, *p* < .001), having begged for food during the past 30 days (F(1) = 12.441, *p* < .001), and being illiterate (F(1) = 5.886, *p* = 0.015).

**Table 6:** Predictors of perceived level of psychological distress

Parameters		*n*	B	Std. Error	Hypothesis Test		Exp(B)	95% Wald Confidence	
					Wald Chi-Squared	Sig.		Lower	Upper
Threshold	Low vs. medium and high levels of distress		−2.638	.3228	66.813	.000	.071	.038	0.135
	Low and medium vs. high levels of distress		−0.576	0.3137	3.376	.066	.562	.304	1.039
Sex	Male	535	−.860	.1293	44.247	.000	.423	.329	.545
	Female	1117	0^a^				1		
Literacy	Yes	104	−.505	.2083	5.886	.015	.603	.401	.907
	No	1548	0^a^				1		
Marital status	Separated/divorced	39	.495	.3729	1.760	.185	1.640	.790	3.407
	Single	78	1.387	.3009	21.244	.000	4.002	2.219	7.217
	Married, polygamous	410	.039	.1360	0.084	.772	1.040	.797	1.358
	Widowed	561	.020	.1537	0.016	.899	1.020	.755	1.378
	Married, monogamous	564	0^a^				1		
Income	Yes	217	−.588	.1481	15.758	.000	.556	.416	.743
	No	1435	0^a^				1		
Handicap	Yes	389	.205	.1459	1.977	.160	1.228	.922	1.634
	No	1263	0^a^				1		
Nutritional needs fulfilled	Yes	767	−.135	.1098	1.508	.219	.874	.705	1.084
	No	885	0^a^				1		
Begging behaviours	Yes	288	.539	.1670	12.441	.000	1.802	1.299	2.500
	No	1364	0^a^				1		
Perceived health status	Good	322	−.932	.1935	23.233	.000	.394	.269	.575
	Average	891	−.777	.1507	26.614	.000	.460	.342	.618
	Poor	439	0^a^				1		
Altered cognitive functioning	No	1095	−.151	.1299	1.345	.246	.860	.667	1.109
	Yes	557	0^a^				1		
Presence of at least one aggressive symptom	No	1435	.300	.1780	2.840	.092	1.350	.952	1.913
	Yes	217	0^a^				1		
Presence of at least one depressive symptom	No	192	−1.043	.1618	41.556	.000	.352	.257	.484
	Yes	1460	0^a^				1		
Presence of at least one psychotic symptom	No	1230	−.220	.1407	2.446	.118	.802	.609	1.057
	Yes	422	0^a^				1		
Age		1652	.017	.0040	19.045	.000	1.017	1.010	1.025

^a^Set to 0 because this parameter is redundant

## Discussion

This exploratory study was a first attempt to document the mental health profile of the poorest in rural Burkina Faso, a very vulnerable sub-population likely to experience mental health distress. Indeed, our participants reported a high proportion of anxious and depressive symptoms, with 40.2% having experienced 10 symptoms or more in the past month. Given that there is no validated tool for the context with a predetermined clinical cut-off, we decided that reporting 10 out of a total of 14 symptoms was an indicator of psychopathology, an assumption that would need to be investigated in future studies.

Our results yielded higher proportions than did previous studies conducted in Burkina Faso. For instance, in their recent study on the prevalence of major depressive episodes in populations living in poor neighbourhoods nearby Ouagadougou, Duthé and colleagues [35] found that 9.0% of their sample (*n* = 2187) reported feeling depressed, sad, or empty, and 7.2% reported having lost interest or pleasure in doing things they usually enjoyed during the past two weeks. While it may not be surprising that people living in extreme poverty conditions in rural settings would report more symptoms than the general population, our results are still alarmingly high. Furthermore, when compared to data from the World Health Survey [39], participants in our sample reported worse perceived general health status, and greater levels of insomnia, sadness, and worries, both in the general population and in the poorest quintile.

Predictors of psychological distress in our study included being a single woman, being illiterate, being elderly, having a poor to average perceived health status, having no income, and having begged for food in the past month. This result is consistent with previous findings in Burkina Faso. In qualitative studies, Roth [40, 41] noted that elders in an urban setting are particularly vulnerable to isolation because of the intergenerational contract that puts the burden of the care of elders on children’s shoulders; our results showed this also seemed to apply in rural settings. Given difficult living conditions and rapid social changes, it appears elders are becoming more numerous and more dependent on their children, who unfortunately are often unable to take care of their aging parents [42]. Other studies in Burkina Faso have identified elders as a population incurring several risks, such as not having their basic needs fulfilled [43], being vulnerable and needing particular help and support [13, 22], experiencing psychological distress and depressive symptoms [43], and being marginalized due to their increasing psychological distress and diminished functioning, which can be related to shame for family members [44].

In her studies, Roth [40, 41] also pointed out that getting older is experienced differently by men and women in traditional settings because they do not carry the same roles and expectations from their entourage, which could lead to different levels of psychological distress. Our results confirmed this observation, as not only were the indigent individuals, as identified by the community-based approach, mainly women (67%), but these women were also more prone to report a high level of distress as compared to men, making them a vulnerable sub-group among the most vulnerable.

Similarly, lack of income and experiencing difficulty accessing food has been reported by Duthé and colleagues [35] as significantly associated with depressive symptoms in Ouagadougou. Nanama and Frongillo [34] found, in a rural setting in Burkina Faso, that food insecurity was an important vulnerability factor associated with anxious, depressive, and somatic symptoms. Duthé and colleagues also found that poor health status predicted the risk of developing depressive symptoms [35], and a qualitative study conducted with indigents and their relatives showed that poor health status was an indicator of extreme vulnerability, even among the least poor households identified as worst-off in rural settings [22].

A large proportion (25.5%) of our respondents reported at least one psychotic symptom, and 65.5% reported having had these symptoms for many years. It is important, however, to point out that the high proportion of psychotic symptoms must be interpreted with caution and within a culturally sensitive framework. The way individuals express distress varies across cultures [45, 46], rendering irrelevant the use of tools or concepts developed in the West to understand mental health needs in a different setting [37, 47, 48]. In fact, many of our respondents refused to answer the questions on psychotic symptoms out of fear of being accused of witchcraft, a socially shared conception of something that, by Western standards, would be considered psychotic/dissociative manifestations [32, 49, 50]. It is thus possible that some individuals did not report these symptoms to avoid stigmatization [51, 52]. It is also possible that other individuals reported psychotic symptoms which are understood by them and their relatives as socially accepted idioms of distress linked to spirit possession [50].

In terms of help-seeking behaviours, our results showed that one-third of respondents who reported any symptom did not seek help or talk to anyone about it. Those who were in poor health, needed to beg for food, and/or had no income were more at risk of not talking to anyone about their symptoms. This may have been because most of our respondents were socially isolated, making it harder for them to seek help. Also, a very small proportion (0.7% for psychotic symptoms and 1.7% for anxious or depressive symptom) did seek help within the formal medical system. Clearly, then, the medical system does not constitute a resource for psychological or mental support for these respondents, while it does for physical health issues. Indeed, between 48.8% and 77.8% of indigents with physical health issues had consulted the modern medical system for curative care [53]. Various factors may explain this discrepancy, including financial barriers, geographical barriers, stigma, lack of mental health literacy, or lack of knowledge about available psychiatric services - factors that are also linked to a lack of trained human resources and to a lack of funding [51, 52, 54–56].

Surprisingly, our respondents also did not appear to have sought the help of traditional healers, who are thought to engage in healing rituals and techniques that fit better with socially shared conceptions of mental health and mental illness [31, 32] than do psychiatric facilities or medical structures. It seems that, in both cases, financial costs and stigma may act as important barriers to care. It is also conceivable that, without these barriers, people experiencing psychotic symptoms would be more inclined to consult a traditional healer due to better compatibility with their beliefs [57]. However, that might not be the case for anxious and depressive symptoms, which might not be understood as “craziness” or spirit possession [32, 49].

### Limitations

We believe an important strength of this study is the fact that we did not rely on a tool developed in the West to collect data and to interpret results. However, our results must therefore be interpreted in light of certain methodological limitations. First, although we developed a context-sensitive tool for this study, the validity and reliability of this instrument needs further assessment to establish clinical cut-offs. Other limitations include: 1) the absence of a comparison group (e.g., general population in the same health districts) and the lack of existing literature to be able to draw conclusions on the impact of living in extreme poverty in this particular context, 2) the absence of questions on perceived social exclusion, and 3) the absence of questions on perceived barriers to care. We also suggest caution in interpreting results due to a possible perception bias among our respondents. Discrepancies between objective measures and self-reported perceptions of health measures might have influenced our data; these effects might also vary in significance between sub-groups of the sample population under study [58].

### Implications for research, policy, and intervention

In line with previous works conducted by our team [19, 38, 59, 60], the current study suggests that the poorest in rural Burkina Faso may face health needs that differ from those of the general population. Hence, specific evidence-based health policies focused on this group’s precarious situation are needed to improve mental health care access. At this stage, further studies are necessary to provide a clear and complex portrait of how psychological distress is expressed and perceived in rural areas. Qualitative interviews should be conducted to explore in greater depth individuals’ perception of mental health needs, the presence of psychopathologies, the absence of help-seeking behaviours, people’s perceptions of formal and informal resources, etc. Qualitative interviews would also provide a better understanding of how different sub-groups among our sample (e.g., women or elders) perceived the questionnaire and how they responded to its questions, which might differ from one sub-group to another due to various factors (e.g., fear of stigma or beliefs about the causes leading to the development of a mental disorder). Ethnographic inquiry could be used to document the context in which those individuals live, which could create meaning that is relevant and useful for the communities, as well as for the tool validation process in that specific setting [37, 61]. Building on these qualitative insights, more quantitative data would then validate a screening tool with a clinical cut-off adapted to the context that could be used by health agencies to identify people in need of psychiatric or psychological services.

Burkina Faso is a country where mental health care is poorly available and accessible. The country has only 0.04 psychiatrists, 0.02 psychologists, and 0.01 social workers per inhabitant, and most are in urban settings [33]. Lacks of trained professionals and of government funding clearly reduce the possibility of universal health coverage. Our results revealed that only a small proportion of the sample reported having sought help from a health professional, in part because of the non-accessibility of care. These results also highlight the importance of family members as the primary resource to which sick individuals can turn to address their concerns and seek help. This additional burden may either render these relatives more vulnerable to economic difficulties and psychological distress themselves, or lead them to socially exclude the sick individuals to avoid these consequences. Consequently, strengthening community cohesion and resilience [62], as well as reducing adverse living conditions and poverty via governmental and non-governmental structures and interventions, should become important public priorities for this specific sub-population.

## Conclusion

In this study, the first to examine mental health outcomes among an indigent population in West Africa, we found that most respondents were reporting psychological distress as well as anxious and depressive symptoms. We consider the absence of help-seeking behaviours directed towards formal or informal resources to be a critical finding, especially since this was also observed among people who had been experiencing psychotic symptoms for many years. Our results highlight the importance of increasing access to mental healthcare in rural areas of the country. We believe future interventions and policies should be adapted to address this vulnerable sub-population, which has specific health needs and is more socially isolated. Services should also be planned according to certain characteristics, such as gender and age, which seem to have a major impact on the way psychological distress is experienced.

## References

[1] World Health Organization (2014). Mental health: a state of well-being.

[2] Whiteford HA, Ferrari AJ, Degenhardt L, Feigin V, Vos T (2015). The global burden of mental, neurological and substance use disorders: an analysis from the global burden of disease study 2010. PLoS One.

[3] Campbell C, Burgess R (2012). The role of communities in advancing the goals of the movement for global mental health. Transcult Psychiatry.

[4] Collins PY, Patel V, Joestl SS, March D, Insel TR, Daar AS (2011). Grand challenges in global mental health. Nature.

[5] Allen J, Balfour R, Bell R, Marmot M (2014). Social determinants of mental health. Int Rev Psychiatry.

[6] Marmot M, Friel S, Bell R, Houweling TA, Taylor S (2008). Commission on Social Determinants of Health. Closing the gap in a generation: health equity through action on the social determinants of health. Lancet.

[7] Compton MT, Shim RS (2015). The social determinants of mental health. Focus.

[8] Das J, Do QT, Friedman J, McKenzie D, Scott K (2007). Mental health and poverty in developing countries: revisiting the relationship. Soc Sci Med.

[9] Patel V, Kleinman A (2003). Poverty and common mental disorders in developing countries. Bull World Health Organ.

[10] Lund C, Breen A, Flisher AJ, Kakuma R, Corrigall J, Joska JA (2010). Poverty and common mental disorders in low and middle income countries: a systematic review. Soc Sci Med.

[11] United Nations Development Programme (2015). The human development report 2015.

[12] Recensement Général de la Population et de l’Habitation (2006). Analyse des résultats définitifs. Thème 5 : caractéristiques économiques de la population.

[13] Ouédraogo S, Ridde V, Atchessi N, Souares A, Kafando Y, Koulidiati JL, Stoeffler Q, & Zunzunegui MV. (in press). Characterisation of the rural indigent population in Burkina Faso: a screening tool for setting priority healthcare services in sub-Saharan Africa. Review submitted to BMJ Open. doi:10.1136/bmjopen-2016-013404 . 10.1136/bmjopen-2016-013405 PMC5640067 28993378

[14] World Bank (2017). The World Bank in Burkina Faso: overview.

[15] Institut national de la statistique et de la démographie; 2015. Profil de pauvreté et d’inégalités. Rapport: Enquête multisectorielle continue 2014. http://www.insd.bf/n/contenu/enquetes_recensements/Enq_EMC/Profil_de_pauvrete_et_d_inegalite_en_2014.pdf. Accessed 28 July 2017.

[16] Ministry of State for Planning, Land Use and Community Development, Ministry of Agriculture and Livestock, & the United Nations System in Burkina Faso. Burkina Faso: accelerating progress toward the MDGs: eradicate extreme poverty and hunger. 2012. http://www.undp.org/content/undp/en/home/search.html?q=burkina+faso%3A+accelarating+toward. Accessed 29 July 2017.

[17] Ridde V, Turcotte-Tremblay AM, Souares A, Lohmann J, Zombré D, Koulidiati JL (2014). Protocol for the process evaluation of interventions combining performance-based financing with health equity in Burkina Faso. Implement Sci.

[18] Ridde V, Rossier C, Soura AB, Bazié F, Kadio K (2014). A community-based approach to indigent selection is difficult to organize in a formal neighbourhood in Ouagadougou, Burkina Faso: a mixed methods exploratory study. Int J Equity Health.

[19] Samb OM. La gratuité des soins et ses effets sociaux: entre renforcement des capabilités et du pouvoir d’agir (empowerment) au Burkina Faso. Université de Montréal: Unpublished doctoral dissertation; 2015. https://papyrus.bib.umontreal.ca/xmlui/handle/1866/11925. Accessed 28 July 2017.

[20] Ridde V, Sombie I (2012). Street-level workers’ criteria for identifying indigents to be exempted from user fees in Burkina Faso. Trop Med Int Health.

[21] Ridde V, Jacob JP (2013). Les indigents et les politiques de santé en Afrique: expériences et enjeux conceptuels.

[22] Kadio K, Ridde V, Samb OM (2014). Les difficultés d'accès aux soins de santé des indigents vivant dans des ménages non pauvres. Sante Publique.

[23] Ridde V, Bonnet E, Nikiema A, Kadio K (2013). A spatial analysis of a community-based selection of indigents in Burkina Faso. Glob Health Promot.

[24] Ouédraogo A, Ouédraogo TL, Sanou PT (2002). Anxiété et dépression chez les personnes vivant avec le VIH en milieu africain à Ouagadougou, Burkina Faso. Psychopathol Afr.

[25] Napon C, Traore S, Niakara A, Ouango G, Ouango A, Kabore J. Les démences en Afrique subsaharienne: aspects cliniques et étiologiques en milieu hospitalier à Ouagadougou (Burkina Faso). Afr J Neurol Sci. 2009;28(1).

[26] Baggaley RF, Ganaba R, Filippi V, Kere M, Marshall T, Sombie I (2007). Detecting depression after pregnancy: the validity of the K10 and K6 in Burkina Faso. Tropical Med Int Health.

[27] Karfo K, Sanou A, Yaogo A, Ouango JG, Ouédraogo A (2009). Aspects épidémiologiques et cliniques de la dépression chez la femme au CHU Yalgado Ouédraogo de Ouagadougou, Burkina Faso. Perspectives Psy.

[28] Karfo K, Thiam MH, Dassa SK, Ouango JG, Ouedraogo A (2007). Aspects psychopathologiques de la dépression du sujet âgé en milieu africain au Burkina Faso. Perspectives Psy.

[29] Napon C, Kaboré A, Kaboré J (2012). La dépression post-accident vasculaire cérébral au Burkina Faso. Pan Afr Med J.

[30] Karfo K, Kiendrebeogo JA, Yaogo A, Ouango JG, Ouédraogo A (2011). Les troubles psychotiques aigus et transitoires au Burkina Faso: aspects épidémiologiques et cliniques à propos de 188 cas. Ann Méd Psychol Rev Psychiatr.

[31] Ouédraogo A, Ouédraogo TL, Traoré A, Sawadogo G, Nebie K, Yougbaré JM (2006). Caractéristiques de la population prise en charge au Service de Psychiatrie du CHU Yalgado Ouédraogo de Ouagadougou (Burkina Faso) de 1990 à 2000. L’Encéphale.

[32] Ouango J-G, Karfo K, Kéré M, Ouédraogo M, Kaboré G, Ouédraogo A (1998). Concept traditionnel de la folie et difficultés thérapeutiques psychiatriques chez les Moosé du Kadiogo. Sante Ment Que.

[33] World Health Organization. Mental Health Atlas 2011: Burkina Faso. http://www.who.int/mental_health/evidence/atlas/profiles/bfa_mh_profile.pdf. Accessed 28 July 2017.

[34] Nanama S, Frongillo EA (2012). Altered social cohesion and adverse psychological experiences with chronic food insecurity in the non-market economy and complex households of Burkina Faso. Soc Sci Med.

[35] Duthé G, Rossier C, Bonnet D, Soura AB, Corker J (2016). Mental health and urban living in sub-Saharan Africa: major depressive episodes among the urban poor in Ouagadougou, Burkina Faso. Popul Health Metrics.

[36] Ridde V, Yaogo M, Kafando Y, Sanfo O, Coulibaly N, Nitiema PA, Bicaba A (2010). A community-based targeting approach to exempt the worst-off from user fees in Burkina Faso. J Epidemiol Community Health.

[37] Sweetland AC, Oquendo MA, Sidat M, Santos PF, Vermund SH, Duarte CS (2014). Closing the mental health gap in low-income settings by building research capacity: perspectives from Mozambique. Ann Glob Health.

[38] Yaogo M, Ridde V, Kafando Y, Kadio K, Mondain N, Bologo E (2012). Enjeux disciplinaires, éthiques et politiques d’une recherche-action concernant l’accès aux soins de santé des indigents au Burkina Faso. La recherche dans des contextes de vulnérabilité: engagement du chercheur et enjeux éthique.

[39] World Health Organization. Burkina Faso - World Health Survey 2003. http://apps.who.int/healthinfo/systems/surveydata/index.php/catalog/20. Accessed 28 July 2017.

[40] Roth C, Alber E, van der Geest S, Reynolds Whyte S (2008). “shameful!” the inverted inter-generational contract in Bobo-Dioulasso, Burkina Faso. Generations in Africa–connections and conflicts.

[41] Roth C (2014). The strength of badenya ties: siblings and social security in old age—the case of urban Burkina Faso. Am Ethnol.

[42] Aboderin I (2004). Decline in material family support for older people in urban Ghana, Africa: understanding processes and causes of change. J Gerontol Ser B Psychol Sci Soc Sci.

[43] Berthé A, Berthé-Sanou L, Konaté B, Hien H, Tou F, Somda S (2013). Les besoins non couverts des personnes âgées en incapacités fonctionnelles à Bobo-Dioulasso (Burkina Faso). Rev Epidemiol Sante Publique.

[44] Ouango JG, Taoko C (2013). La dépression du sujet âgé au Burkina Faso: pourquoi la demande de soins n’est-elle pas formulée?. NPG Neurologie-Psychiatrie-Gériatrie.

[45] Boltanski L (1971). Les usages sociaux du corps. Ann Hist Sci Soc.

[46] Kirmayer LJ (1984). Culture, affect and somatisation, part 1. Transcult Psychiatr Res Rev.

[47] Kirmayer LJ, Young A (1998). Culture and somatization: clinical, epidemiological, and ethnographic perspectives. Psychosom Med.

[48] Kleinman AM (1977). Depression, somatization and the “new cross-cultural psychiatry”. Soc Sci Med (1967).

[49] Olivier de Sardan JP (1994). Possession, affliction et folie: les ruses de la thérapisation. L’Homme.

[50] Mary A (1987). Sorcellerie bocaine, sorcellerie africaine. Le social, le symbolique et l’imaginaire. Cahiers du LASA.

[51] Barke A, Nyarko S, Klecha D (2011). The stigma of mental illness in southern Ghana: attitudes of the urban population and patients’ views. Soc Psychiatry Psychiatr Epidemiol.

[52] Ssebunnya J, Kigozi F, Lund C, Kizza D, Okello E (2009). Stakeholder perceptions of mental health stigma and poverty in Uganda. BMC Int Health Human Rights.

[53] Atchessi N, Ridde V. Enquête sur l’efficacité du ciblage des indigents – rapport partiel sur les caractéristiques des indigents: Unpublished report submitted to the World Bank; 2015.

[54] Andersson LM, Schierenbeck I, Strumpher J, Krantz G, Topper K, Backman G (2013). Help-seeking behaviour, barriers to care and experiences of care among persons with depression in eastern cape, South Africa. J Affect Disord.

[55] Ikwuka U, Galbraith N, Manktelow K, Chen-Wilson J, Oyebode F, Muomah RC, Igboaka A (2016). Ideological vs. instrumental barriers to accessing formal mental health care in the developing world: focus on south-eastern Nigeria. J Health Care Poor Underserved.

[56] Nsereko JR, Kizza D, Kigozi F, Ssebunnya J, Ndyanabangi S, Flisher AJ, Cooper S. & MHaPP research programme consortium. Stakeholder's perceptions of help-seeking behaviour among people with mental health problems in Uganda. Int J Mental Health Syst. 2011;5:5. 10.1186/1752-4458-5-5 PMC3050843 21314989

[57] Stefanovics E, He H, Ofori-Atta A, Cavalcanti MT, Neto HR, Makanjuola V (2016). Cross-national analysis of beliefs and attitude toward mental illness among medical professionals from five countries. Psychiatric Q.

[58] Sen A (2002). Health: perception versus observation. Self-reported morbidity has severe limitations and can be extremely misleading. BMJ.

[59] Atchessi N, Ridde V, Zunzunégui MV (2014). Is the process for selecting indigents to receive free care in Burkina Faso equitable?. BMC Public Health.

[60] Ridde V, Yaogo M, Kafando Y, Kadio K, Ouedraogo M, Bicaba A, Haddad S (2011). Targeting the worst-off for free health care: a process evaluation in Burkina Faso. Eval Program Plann.

[61] Waldram JB (2006). The view from the Hogan: cultural epidemiology and the return to ethnography. Transcult Psychiatry.

[62] Ungar M (2011). The social ecology of resilience: addressing contextual and cultural ambiguity of a nascent construct. Am J Orthopsychiatry.

